# Amplitude-scan classification using artificial neural networks

**DOI:** 10.1038/s41598-018-31021-4

**Published:** 2018-08-20

**Authors:** Kunal K. Dansingani, Kiran Kumar Vupparaboina, Surya Teja Devarkonda, Soumya Jana, Jay Chhablani, K. Bailey Freund

**Affiliations:** 1 Department of Ophthalmology, University of Pittsburgh Medical Center, Pittsburgh, PA USA; 2 L.V. Prasad Eye Institute, Hyderabad, Telangana India; 3 Department of Electrical Engineering, Indian Institute of Technology Hyderabad, Hyderabad, Telangana India; 4 Vitreous Retina Macula Consultants of New York, New York, NY USA; 5 LuEsther T. Mertz Retinal Research Center, Manhattan Eye, Ear and Throat Hospital, New York, NY USA

## Abstract

Optical coherence tomography (OCT) images semi-transparent tissues noninvasively. Relying on backscatter and interferometry to calculate spatial relationships, OCT shares similarities with other pulse-echo modalities. There is considerable interest in using machine learning techniques for automated image classification, particularly among ophthalmologists who rely heavily on diagnostic OCT. Artificial neural networks (ANN) consist of interconnected nodes and can be employed as classifiers after training on large datasets. Conventionally, OCT scans are rendered as 2D or 3D human-readable images of which the smallest depth-resolved unit is the amplitude-scan reflectivity-function profile which is difficult for humans to interpret. We set out to determine whether amplitude-scan reflectivity-function profiles representing disease signatures could be distinguished and classified by a feed-forward ANN. Our classifier achieved high accuracies after training on only 24 eyes, with evidence of good generalization on unseen data. The repertoire of our classifier can now be expanded to include rare and unseen diseases and can be extended to other disciplines and industries.

## Introduction

Optical coherence tomography (OCT) relies on optical backscatter and interferometry to derive a depth-resolved tissue reflectivity map along the axis of illumination^1^. The resulting data can be represented as a plot of log_10_ *reflectivity*^2^ against depth and is referred to as an “amplitude scan” (A-scan) reflectivity-function profile, a nomenclature inherited from ultrasound literature. By traversing the target tissues with the scan beam it is possible to probe reflectivity at different locations which are offset from the primary axis, thereby producing 2-dimensional (cross-sectional) or 3-dimensional (volumetric) maps of log_10_ *reflectivity*^2^ against depth. Current commercially manufactured medical OCT devices are capable of scan speeds of 40,000–100,000 A-scans per second and use light sources centered around 850 nm or 1050 nm to exploit practical windows in the absorption spectrum of water.

**Figure 1:**
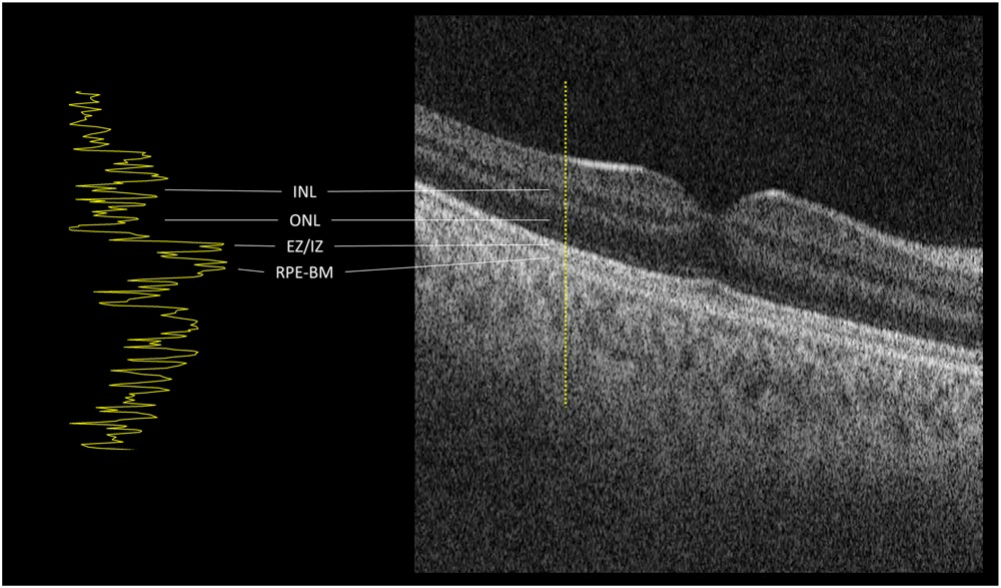
Optical coherence tomography “brightness scan” (B-scan) showing the macular area of a normal subject in cross-section. Image width is 6 mm and depth 1 mm (non-square pixels). Neuronal layers are appreciable as layers of varying reflectivity within the retina. Bruch’s membrane is an important landmark but cannot be resolved separately from the retinal pigment epithelium (RPE) in a normal eye. The foveal depression in the center of the macula is normal. The apparent tilt is due to minimal decentration but is exaggerated by the non-square aspect ratio of the image. An amplitude-scan reflectivity-function profile corresponding to loci along the dotted line is shown in the left panel. (INL: inner nuclear layer. ONL: outer nuclear layer. EZ/IZ: ellipsoid zone and interdigitation zone. RPE-BM: retinal pigment epithelium-Bruch’s membrane complex).

Traditionally, OCT data are rendered as 2-dimensional grayscale images in which pixel brightness codes for log_10_ *reflectivity*^2^; these images are referred to as “brightness scans” (B-scans, Fig. 1). Multiple plane-parallel B-scans taken over a predetermined area facilitate the examination of tissues in cross-sections which are relatively easy for humans to analyze and describe (as in conventional computed tomography or magnetic resonance imaging). If B-scans are acquired with sufficiently close inter-scan proximity they can be assembled into volumetric arrays which may then be re-orientated, re-sliced and re-projected for interactive visualization and intuitive appreciation of 3-dimensional relationships in the data. In ophthalmology, OCT has become a ubiquitous and essential diagnostic and monitoring tool, particularly since the development of effective treatments for common diseases such as age-related macular degeneration, diabetic macular edema and retinal vein occlusion.

An artificial neural network (ANN) is a computational construct consisting of layers of interconnected virtual nodes^2–4^. Each node mimics the behavior of a biological neuron by way of a transfer function that maps an input signal to an output response. Artificial neural networks can be configured to perform information-processing functions and have been shown to have advantages over deterministic techniques for various classification tasks. Artificial neural networks are trained on source data rather than being programmed using parameterized rules. Because training is incremental, building a classifier which generalizes well over unseen data typically requires large datasets.

Recent publications have reported on classifying OCT B-scans or volume scans *in toto* to output the probability of the presence or absence of a particular disease, a binary task in which humans currently set the gold standard^5,6^. However, humans perform this task in a large variety of diagnoses by recognizing a relatively small set of *signatures* (lesion types) occurring in diseases-specific patterns or configurations. We believe that there should be considerable merit in configuring an ANN to perform signature classification as a precursor to disease classification.

In macular disease most disease signatures arise from alterations of the normal lamellar arrangement of ocular posterior segment structures and their corresponding A-scan reflectivity-function profiles. We therefore hypothesize that signature classification should be achievable by an ANN at the level of the *A-scan*, a task which is difficult and unintuitive for humans to perform. This approach has the advantages of data amplification *without* replication or augmentation (because one OCT volume acquisition might yield >100,000 unique A-scans) and generalization across different diseases (including rare diseases).

**Figure 2:**
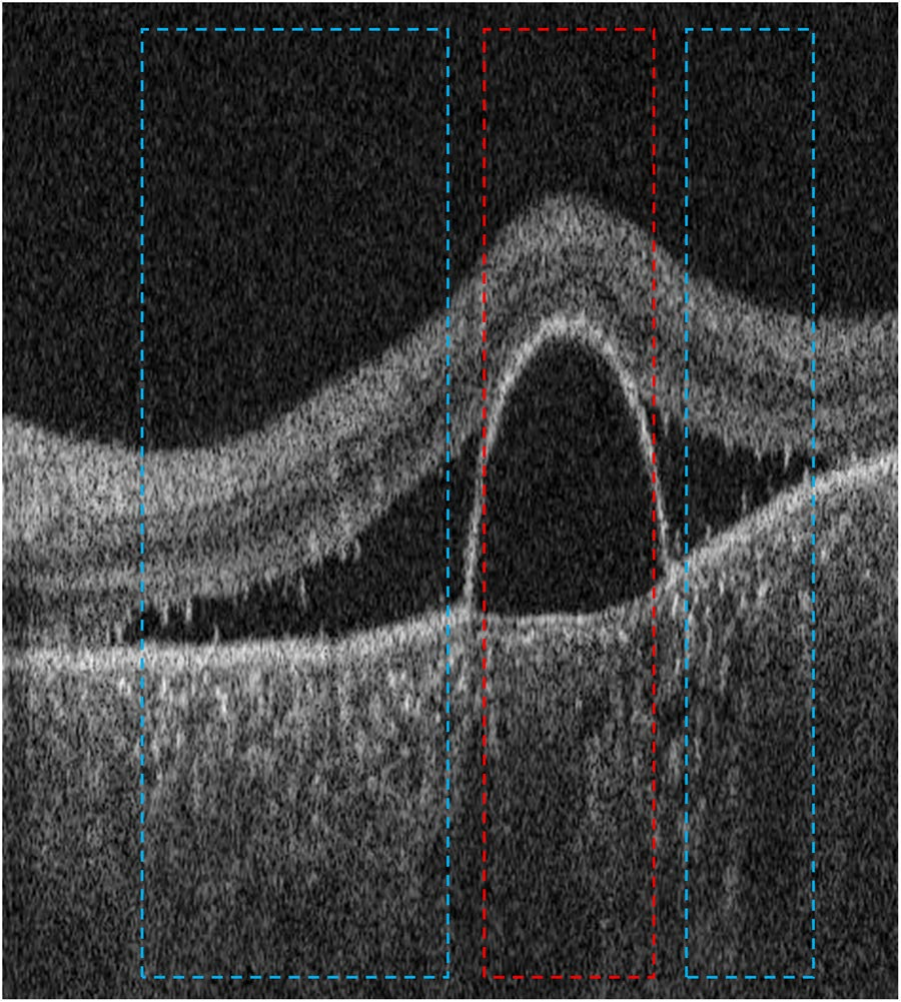
Optical coherence tomography of the left eye in a patient with central serous chorioretinopathy, showing serous pigment epithelial detachment (PED, red box) and retinal detachment (RD, blue boxes). The distinguishing feature is the retinal pigment epithelium which is attached to and indistinguishable from Bruch’s membrane in areas of RD (blue), but detached from Bruch’s membrane in areas of PED (red). Amplitude-scans not falling within colored boxes were left unlabeled and not used for training.

Central serous chorioretinopathy (CSCR) is a disease of the ocular posterior segment, characterized in its early stages by the accumulation of serous fluid in the potential spaces between Bruch’s membrane, retinal pigment epithelium and neurosensory retina. Fluid between Bruch’s membrane and retinal pigment epithelium gives rise to serous pigment epithelial detachment (PED) while fluid between the retinal pigment epithelium and neurosensory retina gives rise to serous retinal detachment (RD). Each of these disease signatures, PED and RD, may occur in isolation, at different locations in the fundus, or may occur in an overlapping configuration (Fig. 2). Each signature produces a distinct alteration of the OCT A-scan reflectivity-function profile and a single A-scan reflectivity-function profile can contain alterations representing either or both signatures.

The purpose of this study was to determine whether OCT A-scan reflectivity profiles of macular disease signatures are classifiable by an ANN, using CSCR as a model disease and using PED and RD as example signatures.

## Results

Twenty-four eyes of 24 patients underwent OCT imaging. These yielded a total of 3,145,728 A-scans, of which 17,500 were human-labeled for serous PED and 399,372 human-labeled for RD.

Confusion matrices for training and testing data are presented (Table 1). On training data, accuracy values were 93.76% (±1.22%), 90.20% (±1.92%) and 94.18% (±1.03%), respectively, for PED, RD and neither. On testing data, accuracy values were 88.04% (±1.71%), 84.27% (±2.20%) and 89.38% (±1.24%), respectively, for PED, RD and neither. Percentages of incorrect predictions were small (Table 1). On unseen data, accuracy values were 78.89 (±1.71), 83.28 (±2.05) and 89.99 (±1.22), respectively, for PED, RD and neither. Sensitivity and specificity values for the classifier in each of the 3 classes are presented as Table 2.

**Table 1:** Confusion matrices obtained after 10-fold Monte Carlo cross-validation for training and testing data (first and second tables, respectively), and for testing on unseen data (lower table).

Predicted → Actual ↓	PED	RD	Neither
**Training**			
PED	93.76 ± 1.22	3.48 ± 0.96	2.75 ± 0.59
RD	4.28 ± 0.85	90.20 ± 1.92	5.51 ± 1.38
Neither	1.01 ± 0.30	4.81 ± 1.02	94.18 ± 1.03
**Testing**			
PED	84.04 ± 1.71	6.66 ± 1.72	5.30 ± 0.96
RD	7.24 ± 1.15	84.27 ± 2.20	8.49 ± 1.64
Neither	2.93 ± 0.63	7.68 ± 1.40	89.38 ± 1.24
**Unseen data**			
PED	78.89 ± 1.71	15.99 ± 4.41	5.11 ± 2.84
RD	4.91 ± 1.01	83.28 ± 2.05	11.79 ± 1.57
Neither	3.01 ± 0.52	6.99 ± 1.26	89.99 ± 1.22

Outcomes are probabilities expressed per cent with standard deviations. Small standard deviations indicate favorable generalization through cross-validation iterations. (PED: pigment epithelial detachment. RD: retinal detachment).

**Table 2:** Sensitivity, specificity values for the classifier during testing in each of the 3 classes: pigment epithelial detachment (PED); retinal detachment (RD); neither PED nor RD.

	Sensitivity	Specificity
PED	0.9164 ± 0.0111	0.9608 ± 0.0052
RD	0.8551 ± 0.0149	0.9366 ± 0.0077
Neither	0.8948 ± 0.0117	0.9358 ± 0.0067

**Figure 3:**
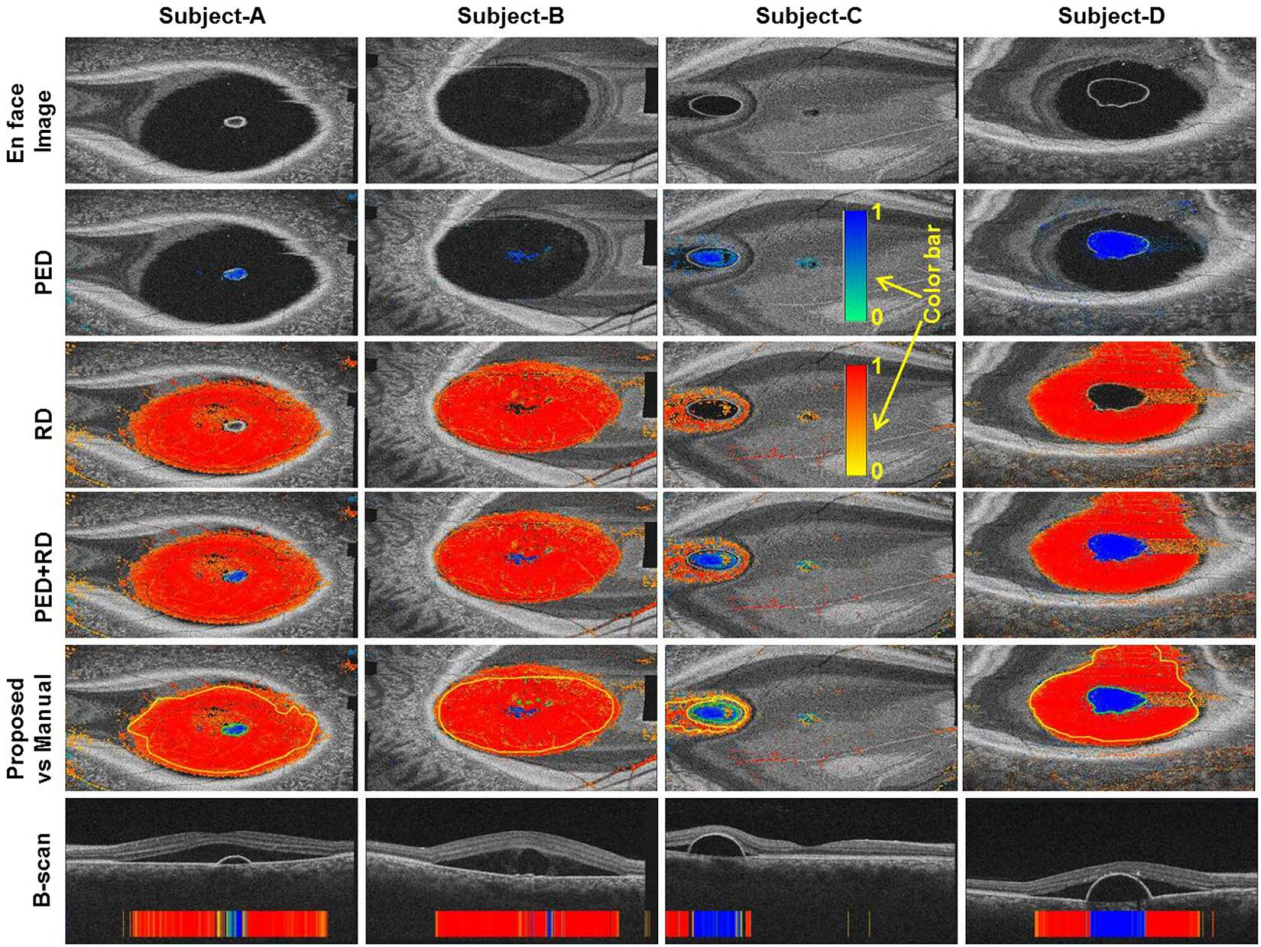
En face (coronal) projections of optical coherence tomography (OCT) scans of 3 patients with central serous chorioretinopathy, with overlaid classification data. First row: En face OCT sections which best illustrate the distribution of retinal detachment (RD) and pigment epithelial detachment (PED) in each eye. Second row: Shaded pixels (according to color bar) represent the probabilities, as determined by the trained classifier, of respective amplitude-scans representing PED. Third row: Shaded pixels (according to color bar) represent the probabilities, as determined by the trained classifier, of respective amplitude-scans representing RD. Fourth row: Combined RD and PED maps. Fifth row: Combined probability maps for RD and PED, as determined by the trained classifier, with boundaries of human-label maps overlaid in yellow (RD) and green (PED) for comparison. Bottom row: Selected OCT B-scans illustrating RD and PED, with classifier output demonstrated per amplitude scan in color.

Figure 3 compares the en face distributions of A-scans for each signature as labeled manually with those labeled by the classifier, in 3 representative eyes. Qualitatively, close agreement is appreciated between human- and classifier-labeled signature distributions. Notably, where the human had been conservative, especially at the margins of a region of PED or RD, the classifier generalized beyond the human-labeled signature boundaries and classified unseen A-scans representing even small degrees of PED and/or RD. In these regions of subtlety, A-scans representing PED and RD are less distinguishable from those representing neither signature, and the decrease in classifier sensitivity is represented by decreased pixel brightness at the signature margins in the classifier’s heat map. Some A-scans were labeled by the classifier as false positives for PED or RD. Spurious labeling occurred more frequently over prominent retinal blood vessels.

## Discussion

Developments in computational technology at accessible cost have led to increased interest in adopting machine learning to perform tasks which would be difficult or impossible to perform using procedural or deterministic methods. Training of machine learning systems is also made possible by the availability of large amounts of digitized and coded data which mankind have accumulated over recent decades.

In conceiving this study we recognized that OCT data are orientated consistently across datasets, due to the inherently directional nature of the modality and the constraints imposed by having to image through the pupil. We sought to determine whether OCT A-scan reflectivity-function profiles of the macula exhibit sufficient specificity for the anatomical signatures they represent to be classified accurately by a feed-forward ANN.

Our tool chain and classifier achieved high accuracy in the two classes with the greatest numbers of training samples, with good generalization reflected by small standard deviations during cross-validation (Table 1). The findings suggest that OCT A-scan reflectivity profiles are indeed signature-specific and classifiable. Amplitude-scans of PEDs were fewer in number than those of RD but still achieved favorable metrics. The findings raise several points for discussion and many avenues for refinement and further inquiry.

Image classification is frequently approached using a convolutional neural network, a type of ANN in which the configuration of nodes and connections (topology) is inspired by the receptive fields and hierarchical processing understood to occur in sensory systems in living creatures^7–9^. Convolutional neural networks have found roles in many applications such as computer vision and speech and language recognition and abstraction, and are also employed in medical image classification. Gargeya and Leng have used a convolutional neural network to perform binary classification of color fundus photographs as normal or consistent with diabetic retinopathy, with sensitivity and specificity of 94% and 98%, respectively^10^. Their dataset consisted of 75,137 expert-labeled fundus photographs representing all stages of diabetic retinopathy, including absent retinopathy. Lee and associates recently published results of binary classification of OCT images as either normal or representing age-related macular degeneration, using a database of approximately 50,000 labeled images in each class, and reporting sensitivity and specificities around 93%^11^. Although the investigators in each of these studies were able to perform secondary analyses to identify the features in the images which made the images diagnostic, each study was confined to its respective overall disease of interest.

Recently, Schlegl and associates have applied deep-learning to detect and quantify subretinal fluid and intraretinal fluid in B-scan images of eyes with neovascular age-related macular degeneration^12^. Their study demonstrates the value of classifying a disease signature but because their analysis is performed at the level of the B-scan, they have still relied on a relatively large dataset (1,200 patients) and used machine learning to replicate a task in which human performance remains the gold standard.

There are two significant theoretical advantages to classifying A-scans for signature rather than B-scans or volume scans for diagnosis. The first is that a large number of unique, signature-specific A-scans can be extracted from a single B-scan or volume scan, thereby greatly increasing the practicality of training a classifier with data from fewer patients than with other approaches. The second theoretical advantage is that an A-scan classifier should be able to function over a broad range of diseases, including rare ones for which sufficiently large numbers of scans may not be available for disease-specific training.

In the study of macular disease our next objectives are to increase the repertoire of signatures to include internally reflective PED, subretinal hyperreflective material, outer retinal atrophy, cystoid macular edema, intraretinal hyperreflective foci, epiretinal membrane and “thick” and “thin” choroid, a list which in different permutations of its elements describes the majority of macular diagnoses known to humans. The main limitation of our technique that manual labeling of signatures is labor intensive, especially when signatures are numerous, subtle or non-contiguous (e.g. pseudodrusen, a particular discrete, multifocal form of subretinal hyperreflective material). This might be overcome by using *un*supervised learning to cluster A-scans into automatically determined classes which can be labeled *post hoc*. Such an approach might also reveal OCT signatures which are as yet undiscovered, or the significance of which has not yet been established.

We envisage that an A-scan classifier can be incorporated into the viewing and analysis software which accompanies commercially available OCT devices, enabling signatures and other features of interest to be labeled immediately after scan-acquisition. For diagnosing disease, the possibility remains of classifying en face signature probability maps in a second stage convolutional neural network.

The potential scope this work is not limited to OCT imaging of the ocular posterior segment. Optical coherence tomography has utility in a large range of disciplines outside ophthalmology, medicine and even biology^13–15^. Classification of A-scan reflectivity-function profiles may be important in studying extraocular tissues such as coronary vessels and alimentary tract epithelium. In scenarios where OCT is not frequently used, such as *ex vivo* imaging or non-invasive imaging of bio-engineered tissues, the potential for automated signature labeling at the time of scan acquisition may make the adoption of OCT in these wider contexts highly appealing. Generalizing beyond OCT and biology, the concept of classifying A-scans is extendable to any scenario in which lamellar structures are imaged by analyzing *reflected energy*, either by interferometry or by traditional pulse-delay-echo techniques, as for example in reflection seismology.

The main limitation of this study is the small number of eyes imaged. In order to label data for supervised learning with minimum labor, an en face label painting approach was adopted (Methods). This necessitated the use of *volumetric* scans acquired on a relatively new swept-source OCT device, on which a limited number of subjects had been scanned. Nevertheless, the A-scan yield was sufficient to train a neural network in a 3-class problem and to demonstrate that A-scan reflectivity-function profiles are sufficiently specific to certain disease signatures to be classified for further analysis. Further work will be directed towards increasing the number of classes and the range of devices, and quantifying generalizability.

## Methods

This study was conducted under a protocol approved by the ethics committee at the L.V. Prasad Eye Institute, Hyderabad, India, and complied with the tenets of the declaration of Helsinki. All patients provided informed consent for clinical imaging and analysis. Scan data were anonymized for analysis, as required by the Health Insurance Portability and Accountability Act of 1996.

Patients with acute central serous chorioretinopathy were identified and included for study if they had undergone swept-source OCT imaging as part of their clinical care and if either eye exhibited PED, RD or both. Patients with other disease signatures, such as neovascularization, were not enrolled. The OCT volume scans had been acquired using a “Triton” DRI OCT device (Topcon Medical, Oakland, NJ) over a 6 mm × 6 mm area centered at the fovea. Each volume scan consisted of 256 B-scans, each of which consisted of 512 A-scans. Each A-scan consisted of 992 pixels and was orientated approximately perpendicular to the macular lamellae. The DRI OCT is a swept source OCT device which uses a tunable light source centered at 1050 nm and scans at 100,000 A-scans per second.

**Figure 4:**
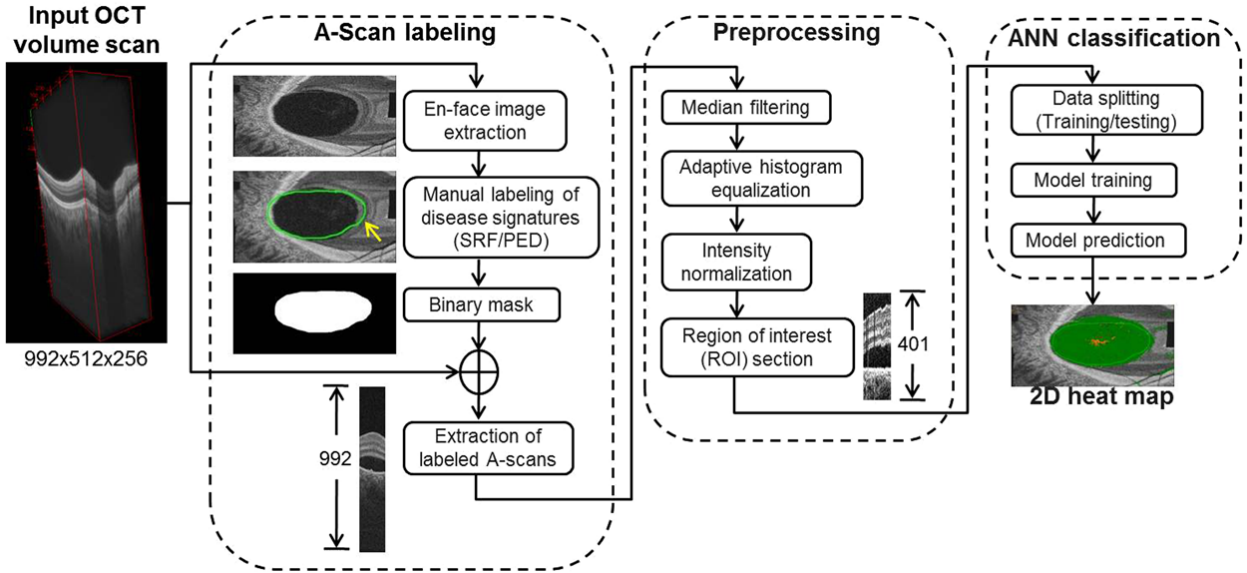
Schematic representation of steps in image processing, labeling and classification. (OCT: optical coherence tomography. A-scan: amplitude scan).

A schematic describing our methods is presented as Fig. 4 and the steps are described in detail as follows.

### Amplitude-scan labeling

**Figure 5:**
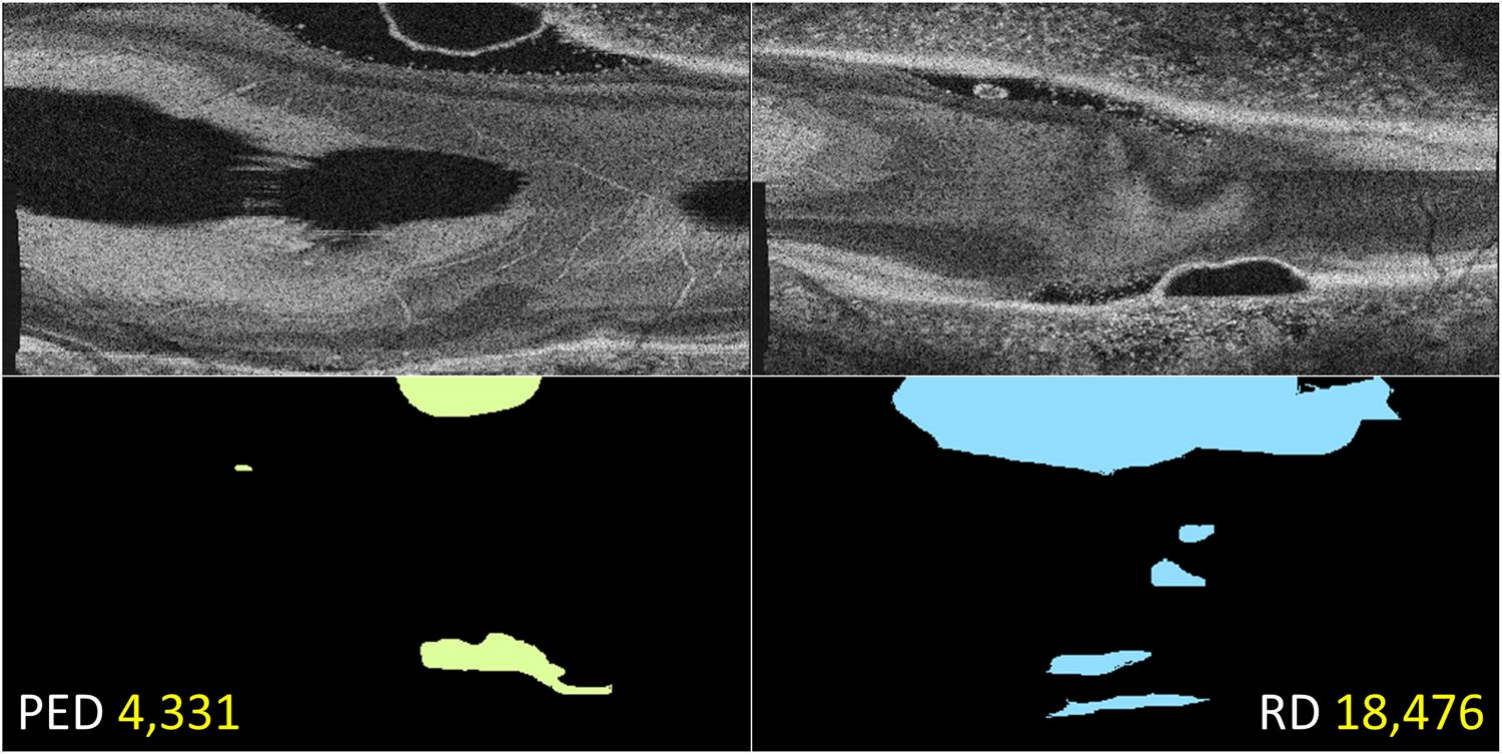
Amplitude-scan labeling for disease signature, by a human. En face (coronal) optical coherence tomography (only 2 slices included, top row) shows the extent of pigment epithelial detachment (PED) and retinal detachment (RD). Amplitude-scans are labeled for PED and RD by scrolling through the entire en face image stack and using synchronized cursors in ImageJ to draw on blank canvases. Numbers in yellow represent the number of signature-specific A-scans labeled for each respective signature.

Each volume scan was reoriented and re-sliced to be viewed from an en face perspective so that the distribution of PED and RD in the fundus could be appreciated (ImageJ 2, Fiji Life-line, http://fiji.sc)^16^. By scrolling through the stack of en face segments, and using synchronized cursors, the distributions of PED and RD were drawn manually on a blank canvas to produce en face distribution maps of each signature in each eye, thereby labeling each A-scan which described a signature of interest (Fig. 5). Where there was uncertainty as to the quality of the A-scan in representing its signature(s) of interest, the A-scan was regarded as “equivocal” and therefore neither labeled nor used for training and validation; equivocal A-scans were used, however, for testing. Examples of such A-scans were those representing subretinal fibrin, very shallow RD or the steep borders of dome-shaped PEDs (Fig. 2). Amplitude-scans outside the optic disc which the human labeler was confident did not represent either PED or RD were labeled as representing neither of the signatures.

### Pre-processing

The scan data of each eye consisted of 256 B-scans, each of 512 pixels (wide) by 902 pixels (deep). The OCT volume data were preprocessed to improve consistency for training using a number of transformations. Transformations designed to operate on 2-dimensional images were applied at the B-scan level while others were applied on a per A-scan basis.

The B-scan images were initially passed through a median filter to remove noise by replacing each pixel with the median of neighboring pixels. Following this a contrast limited adaptive histogram equalizer was applied to improve contrast by redistributing lightness values and limiting noise amplification.

Feature extraction was performed to crop the OCT data to include only relevant anatomy, so that the complexity of the classifier could be minimized but A-scan signature specificity could be maximized. Since the signatures of interest in acute central serous chorioretinopathy occur adjacent to the retinal pigment epithelium, which is typically the most reflective layer in an OCT scan, the first step was to estimate the location of the retinal pigment epithelium in each B-scan using a 40-pixel neighborhood maximum mean intensity search. Each A-scan was then cropped to include 300 pixels anterior to the retinal pigment epithelium and 100 pixels posterior (deep) to the retinal pigment epithelium. The resulting 401-length vector from each 992-length A-scan was treated as the feature vector for the classifier.

All the labeled A-scans were extracted from each study eye, retaining anatomical positional information, and processed as above to yield vectors of length 401. All features were normalized so that the mean and variance of each feature was 0 and 1, respectively, with the aim of ensuring that no particular feature would bias the classifier.

### Classification

A feed-forward ANN was employed to build a three-class A-scan reflectivity-function profile classifier (PED, RD, neither) with the following parameters: network structure, 400–200–75–10–3; optimizer, Adam; learning rate, 0.001; hidden and output layer activation function, sigmoid; loss function, binary cross-entropy.

Classification involved 2 phases: training and testing. All the human-labeled A-scans from the 24 eyes were used for training and testing (80:20 split). During training, equal numbers of features from each class were sampled to avoid biasing the classifier with unequal feature numbers between classes. Since PED A-scans were much fewer than RD A-scans (as expected in CSCR, see Results), training uniformity was maintained by random selection of 14,000 A-scans representing each signature and 14,000 A-scans representing neither signature. Classifier training was performed by backpropagation, which utilizes gradient descent.

Cross-validation was performed using a Monte Carlo strategy over 10 iterations. In each iteration the feature collection was split randomly into training, validation and testing datasets and after the 10 iterations the standard deviations of classifier performance indices were taken as metrics for generalization.

The ANN was implemented in Python version 2.7 using the Keras module (https://github.com/fchollet/keras). The trained model was tested on 12 unseen eyes, an data set consisting of 5,187 A-scans of PED, 73,241 A-scans of RD and 897,063 A-scans of neither PED nor RD.

### Performance analysis

The efficacy of our proposed methodology was assessed quantitatively by preparing confusion matrices to demonstrate the classifier’s performance on training and testing data.

To visualize the performance of the classifier the anatomical positional data of feature vectors was used to plot 2-dimensional en face maps with pixel color coding for signature probability. These probability distribution maps were superimposed on human-label maps and en face OCT projections for direct visual comparison and qualitative assessment.

## Data Availability

The datasets generated and analysed during the current study are not publicly available, since they constitute patient biometric data, but are available from the corresponding author on reasonable request.
